# Active exoskeleton reduces erector spinae muscle activity during lifting

**DOI:** 10.3389/fbioe.2023.1143926

**Published:** 2023-04-25

**Authors:** Tobias Walter, Norman Stutzig, Tobias Siebert

**Affiliations:** ^1^ Motion and Exercise Science, University of Stuttgart, Stuttgart, Germany; ^2^ Stuttgart Center for Simulation Science, University of Stuttgart, Stuttgart, Germany

**Keywords:** exoskeleton, occupational safety, musculoskeletal disorders, lower back, Cray X, electromyography, muscle activity, perceived exertion

## Abstract

Musculoskeletal disorders (MSD) are a widespread problem, often regarding the lumbar region. Exoskeletons designed to support the lower back could be used in physically demanding professions with the intention of reducing the strain on the musculoskeletal system, e.g., by lowering task-related muscle activation. The present study aims to investigate the effect of an active exoskeleton on back muscle activity when lifting weights. Within the framework of the study, 14 subjects were asked to lift a 15 kg box with and without an active exoskeleton which allows the adjustment of different levels of support, while the activity of their *M. erector spinae* (MES) was measured using surface electromyography. Additionally, the subjects were asked about their overall rating of perceived exertion (RPE) during lifting under various conditions. Using the exoskeleton with the maximum level of support, the muscle activity was significantly lower than without exoskeleton. A significant correlation was found between the exoskeleton’s support level and the reduction of MES activity. The higher the support level, the lower the observed muscle activity. Furthermore, when lifting with the maximum level of support, RPE was found to be significantly lower than without exoskeleton too. A reduction in the MES activity indicates actual support for the movement task and might indicate lower compression forces in the lumbar region. It is concluded that the active exoskeleton supports people noticeably when lifting heavy weights. Exoskeletons seem to be a powerful tool for reducing load during physically demanding jobs and thus, their use might be helpful in lowering the risk of MSD.

## 1 Introduction

### 1.1 Prevalence of musculoskeletal disorders

Musculoskeletal disorders (MSD) are the most common work-related health problem in the EU (European Agency for Safety and Health at Work, 2019b). Work-related means that they are caused or at least worsened by work or the immediate work environment. More than half of the European workers suffer from some form of MSD. Back pain is the most commonly reported one (European Agency for Safety and Health at Work, 2019b). The aetiology of MSD seems to be multifactorial (European Agency for Safety and Health at Work, 2019b). Occupational factors are one relevant element and according to estimates, about 37% of non-traumatic low back pain can be attributed to them (Punnett et al., 2005). Among others, lifting objects is one of the physical factors at the workplace that is assumed to have a causal relationship with back pain (National Institute for Occupational Safety and Health, 1997). Still, about a third of the workers in the EU have to carry or move heavy loads during a quarter or more of their working time (European Foundation for the Improvement of Living and Working Conditions, 2017). In addition to employers’ responsibility towards the health of their employees, MSD result in high costs for companies, which is why preventive actions should not only be in the interest of the workers concerned but also in the interest of the employers (Thiehoff, 2002). In Germany, for example, MSD and connective tissue disorders were estimated to be responsible for 17.2 billion euros in loss of production only based on labour costs in 2016 (European Agency for Safety and Health at Work, 2019b). The demographic change and the growing shortage of skilled workers exacerbate the situation at the workplace and increase the significance of the challenge to keep employees healthy in the work process for as long as possible (Schick, 2018).

### 1.2 Back-support exoskeletons and their biomechanical evaluation

A potential tool to reduce the load in physically demanding jobs might be mechanical structures worn on the human body to support the musculoskeletal system of the user during certain movements and postures, so-called exoskeletons (Kaupe and Feldmann, 2021). Exoskeletons can be divided regarding the body segments they support (e.g., the lower back, shoulder or leg) or how they work (e.g., passive or active). While passive systems work with mechanical structures like springs which store energy during certain movement sequences and release it to another point in time, active exoskeletons use actuators such as electromotors. The European Agency for Safety and Health at Work (EU-OSHA) considers exoskeletons potentially helpful in mobile workplaces where few ergonomic measures can be implemented (European Agency for Safety and Health at Work, 2019a). Possible fields of application are especially where automation is not yet feasible, among others due to the flexibility required within the scope of the tasks which have to be performed (Sylla et al., 2014).

To assure that the use of industrial exoskeletons is in favour of the health and contentment of the workers, the evaluation of exoskeletons is a major step. The research interest in this field is considerably growing, which is evident from the substantial increase in related publications within the last few years (Koopman et al., 2019; Lazzaroni et al., 2019; Poliero et al., 2020; Kermavnar et al., 2021; Pesenti et al., 2021). A parameter often considered in common biomechanical investigations is muscle activity measured via electromyography (EMG) (Müller et al., 2012; Stutzig and Siebert, 2016; Laßberg et al., 2017). When using an exoskeleton, a reduction in muscle activity in the intended area is generally interpreted as a relief and thus as a confirmation of the functioning of the respective exoskeleton (Kermavnar et al., 2021). Regarding exoskeletons for supporting the lower back, which are mainly designed for dynamic lifting and static holding activities, according to a review from Kermavnar et al. (2021), the *M. erector spinae* (MES) is the most frequently investigated muscle group. In 22 out of 27 studies which investigated the influence of exoskeletons on back-muscle activity, a significant reduction was found when using an exoskeleton compared to performing the same task without exoskeleton. The average reduction regarding the activity of the MES was 25% for active exoskeletons (values between 6% and 48% of reduction have been reached) and was somewhat lower with 18% for passive exoskeletons (values between 6% and 35% of reduction have been reached). Irrespective of this, both, active and passive exoskeletons, have advantages and disadvantages and do not have to mutually exclude each other, but could rather be seen as complementary. Whereas passive back exoskeletons are in general lighter and cheaper, active ones seem to have a higher potential of reducing physical loads (Looze et al., 2016; Ali et al., 2021). Pesenti et al. (2021) conclude that passive systems may be more adequate when relatively low power delivery is required throughout the whole working day, as in the case of static forward bending, whereas active systems may be preferred when bursts of high power are required. A major advantage of active exoskeletons is that the provided assistance can be modulated via the exoskeleton’s control system regulating the actuators. This facilitates the adaptation of active exoskeletons and their support to the given conditions and the individual preferences of the user, thus enabling a more versatile operation.

### 1.3 Contribution of this study

An example of an active back-support exoskeleton allowing the user to manually adjust the level of support is the so-called Cray X (German Bionic Systems GmbH, Germany) which can assist in lifting tasks by augmenting hip extension. Via a user interface consisting of a display and a rotary and push knob, the level of support can be varied gradually between 0% and 100%. Since, to our knowledge, there is no study focused on the biomechanical examination of the Cray X (4th generation), the question arises whether the use of this exoskeleton leads to a reduction in the activity of the spinal extensors, and in particular, whether a higher level of support necessarily leads to greater muscle relief.

To investigate these questions, subjects were asked to lift a load under all eleven available exoskeleton support levels (0%–100% in 10% steps) as well as without exoskeleton. Subsequently, the following three main conditions: 1) without exoskeleton, 2) inactive exoskeleton (0% support level) and 3) active exoskeleton (100% support level) were evaluated for statistically significant differences regarding the muscle activity of the MES. Furthermore, it was investigated if there is a correlation between the used support level (0%–100%) and muscle activity. To be able to make statements about whether potential effects are noticeable to the user, the subjectively perceived rate of exertion was additionally investigated under each condition.

## 2 Methods

### 2.1 Participants

Fourteen young and healthy adults (sex: three females and eleven males, age: 22.3 ± 1.1 years, height: 177.7 ± 7.2 cm, weight: 71.9 ± 10 kg) participated in the study. All of them had no or very little experience with the use of exoskeletons. The subjects were informed about possible risks of the experiments and gave their written consent. The study was approved by the University of Stuttgart Ethics Committee (AZ. 22-002) and was conducted in accordance with the latest declaration of Helsinki.

### 2.2 Exoskeleton

**FIGURE 1:**
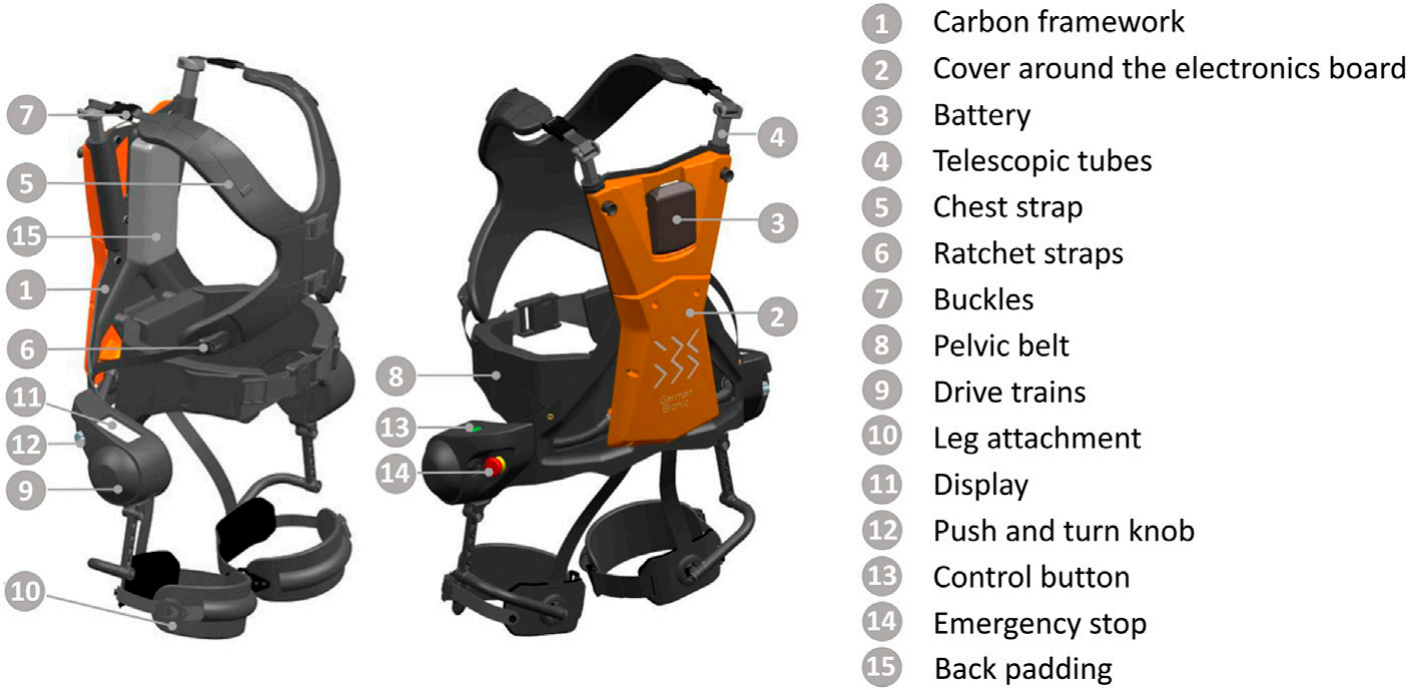
Labelled 3D model of the active exoskeleton Cray X in its fourth generation (German Bionic Systems GmbH, Germany).

**FIGURE 2:**
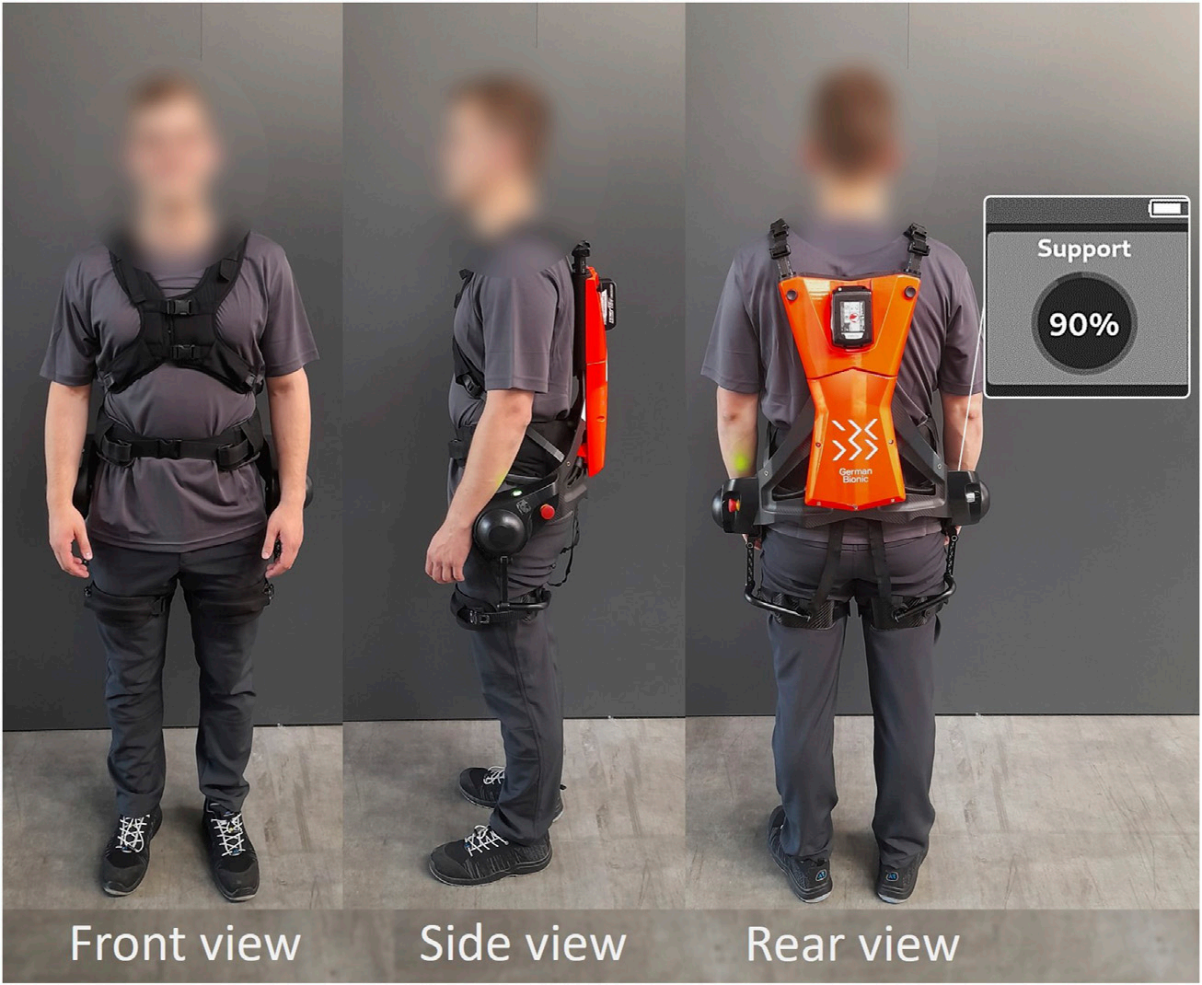
Subject wearing the Cray X exoskeleton.

The commercially available active exoskeleton Cray X in its fourth generation (Figure 1) was used in this study. The exoskeleton weighs about 8 kg and is worn like a hiking backpack (Figure 2), with a pelvic belt supporting most of the dead weight of the exoskeleton on the user’s iliac crest. In addition to the pelvic belt, there is a fixation on the user via a chest strap and two leg connections. The torso inclination and movement are measured by built-in inertial measurement units. The exoskeleton generates an extension torque at the level of the hip through two battery-powered electromotors in the sagittal plane. The torque is transmitted to the body via the adjustable connections mentioned before. The generated torque depends on I) the selected work mode (static vs. agile), II) the basic device parameter settings (allow targeted adaptation, e.g., to the given conditions and individual preferences) and III) the movement velocity of the subject. Since a speed-dependent control is used, the generation of torque is initiated by movement impulses. Regarding the basic device settings II), the relevant parameter for adjusting the level of torque generated when lifting and therefore straightening the back is the so-called support level. Even though the exoskeleton offers the possibility to influence the amount of torque generated in the lowering movement too, utilizing a further parameter (called counterforce), this was not exercised and the parameter was set to zero throughout. For reasons of clarity and to obtain differentiated results, it was decided to focus on one parameter only. Thus, there was no torque acting against the upper body during the lowering phase.

### 2.3 Experimental protocol

**FIGURE 3:**
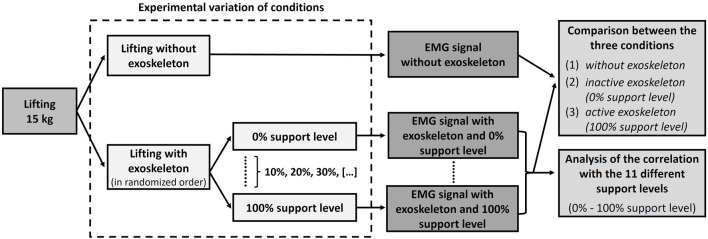
Schematic structure of the study design with regard to the analysis of the impact of exoskeleton use on muscle activity.

**FIGURE 4:**
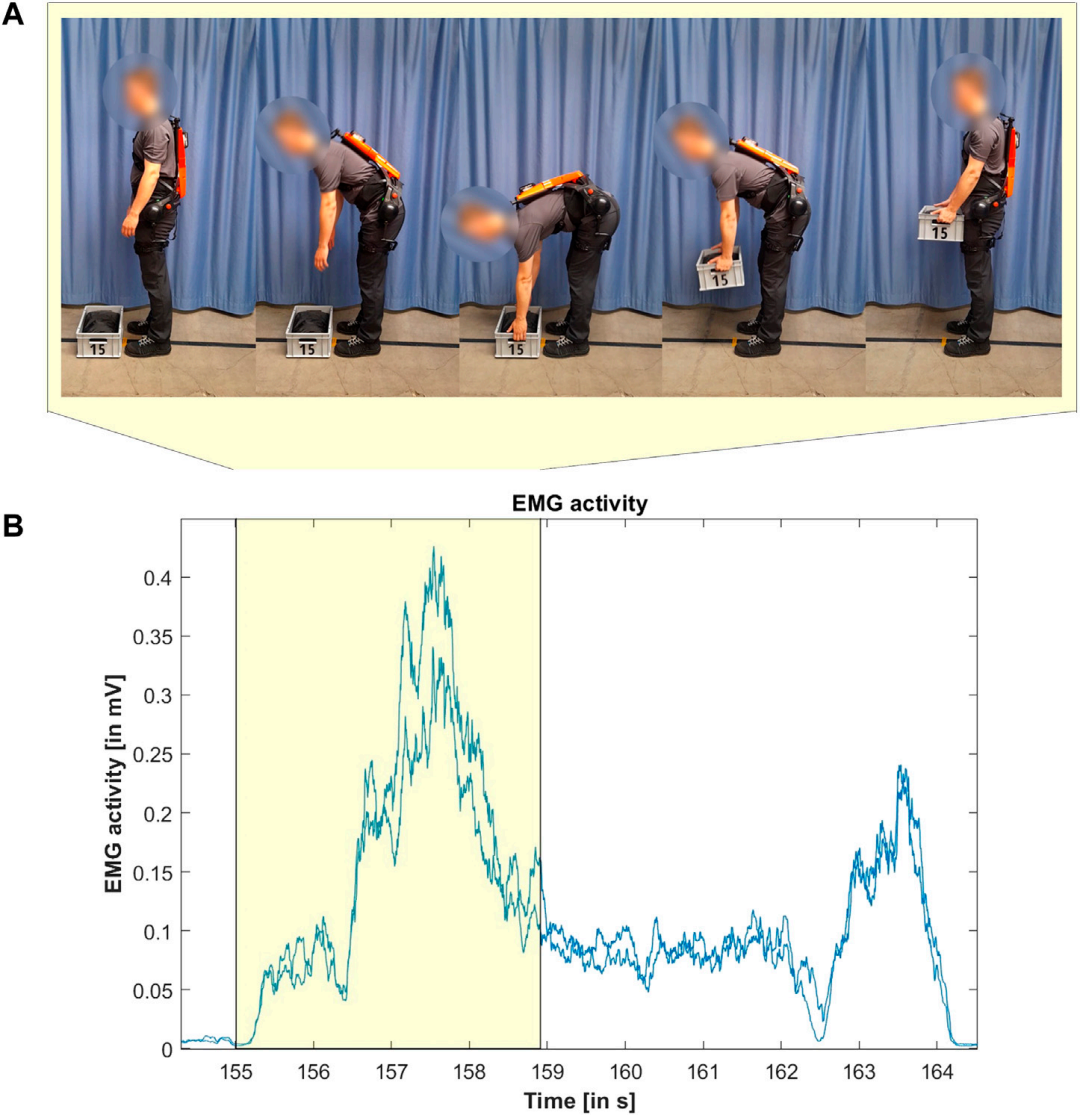
Conducted lifting movement ((A)—upper row) and exemplary corresponding EMG signal (blue lines) of left and right *M. erector spinae* ((B)—lower row).

To examine the impact of the main lifting conditions 1) without exoskeleton, 2) inactive exoskeleton (0% support level) and 3) active exoskeleton (100% support level) as well as the gradually adjusted support level of the exoskeleton on MES activity, the following experimental protocol has been performed (Figure 3). The participants were asked to lift a 15 kg box (40 × 30 × 22 cm transport box with handle openings) from the ground to standing straight (Figure 4A) using the stoop lifting technique (i.e., knees straight and hip bent). Twelve different box lifting experiments (1 without exoskeleton and 11 with exoskeleton and support level varied between 0% and 100% in 10% steps) had to be executed by each participant. Each experiment consisted of 4 single lifts. All participants started without the exoskeleton. The order of the different lifting experiments with the exoskeleton, however, was randomized and the participants did not know under which support level they were lifting, as the necessary setting adjustments were conducted by the investigator. There was a pause of 20 s between the single lifts and a further break of 1 min between the different experiments. No specifications were made for the lifting speed in order to allow a natural execution of the lifting movement. Before the experimental protocol started, the participants got an introduction to the use of the exoskeleton and a short, 10-min testing trial was conducted for familiarization.

### 2.4 Data recording

Bipolar surface EMG measurements were conducted on the left and right MES (specifically on the M. longissimus). Therefore, the skin was prepared by shaving, abrading, and cleaning with alcohol. Two self-adhesive Ag/AgCl electrodes were placed two finger width lateral from the spinous process of the first lumbar vertebra in line with the muscle fibre direction afterwards, following the SENIAM guidelines (Hermens, 1999). Generally, at this location, sufficient space is available between the pelvic belt and the back padding of the exoskeleton (see Figure 1). In some cases, it was necessary to place the electrodes higher to prevent the pelvic belt from exerting force on the electrodes during lifting. The EMG data were recorded using the BTS Bioengineering FREEEMG 1000 system (BTS Bioengineering S.p.A., Italy) with a sample rate of 1,000 Hz and stored on a computer.

To be able to normalize the EMG data later, maximum voluntary contractions (MVC) have been conducted (Siebert et al., 2022). Therefore, the participants laid on their stomach and tried to lift their upper body as much as possible while the investigator manually fixed their legs on the floor and applied resistance against their upper back. The participants performed this static exercise three times, each time holding on for 3 s. The highest value of the processed EMG data was taken to normalize the data obtained while lifting afterwards.

The participants were filmed with a camera (30 Hz) to document the start and the end of every lifting movement. Using the synchronised video data, it was possible to extract the intervals from the EMG data during which the lifting movements took place. For the start, the picture was taken in which a movement in the sense of bending forward of the upper body to reach the box was first recognisable. The movement was defined as finished as soon as the subjects stood upright again, holding the box in their hands (Figure 4A). All relevant intervals in the EMG data (Figure 4B, exemplary section highlighted in yellow) were visually checked for measurement artefacts, e.g., due to contact of the exoskeleton with the electrodes, and sorted out if necessary.

Additionally, the participants were asked about their rating of perceived exertion (RPE) after completing all four consecutive lifts of each experiment. They verbally categorized their overall perceived load level using a 15-level Borg scale (Borg, 1998) reaching from rating 6 (no exertion at all) to 20 (maximal exertion).

### 2.5 Data processing

For each experiment, only the EMG data of the last three of four lifts have been evaluated. This procedure was chosen since, as already mentioned before, the subjects did not know under which level of support they were performing the movements when using the exoskeleton to not influence their subjective perception of exertion. As a result, they could not anticipate how strong the torque of the exoskeleton would be and how much force they would have to apply themselves during the first repetition. Therefore, the first lifting movement in each experimental condition served as a preparation trial for the following lifts.

The raw EMG data were processed using Matlab^®^ R2020b (Massachusetts: The MathWorks Inc.). To filter low-frequency movement artefacts, a second-order high-pass Butterworth filter with a cut-off frequency of 10 Hz was applied. Furthermore, the data have been full-wave rectified and smoothed with a moving-average filter of 150 ms. Afterwards, the EMG data were normalized if necessary for the respective part of the evaluation and presented as % MVC. The Root-Mean-Square (RMS) of the EMG activity was examined and averaged for the left and right MES over each lifting period before the mean was calculated over all three evaluated repetitions under each condition for every participant. If the data from one of the three repetitions had to be eliminated due to measurement artefacts, the mean value was calculated analogously over the RMS of the two remaining repetitions. If two repetitions were affected by artefacts and therefore no averaging was possible, the data of the participant recorded under the respective condition was omitted. The same applies, of course, to trials in which all three repetitions were affected by measurement artefacts.

### 2.6 Statistical analysis

For further analysis, the data regarding the muscle activity were tested for normal distribution using Q-Q diagrams and the Shapiro-Wilk test. All data were normally distributed. Mauchly test was used to confirm the condition of sphericity of the data. A one-way repeated measures analysis of variance (rmANOVA) was used to compare the three main lifting conditions 1) without exoskeleton, 2) inactive exoskeleton (0% support level) and 3) active exoskeleton (100% support level) in regard to differences in MES activity. In the case of main effects, *post hoc* analyses were performed using the Bonferroni test. The alpha level was set at 5%.

To check for a relationship between the support level of the exoskeleton and the observed MES activity, Spearman correlations were used. The correlations were determined for the subjects individually as well as for the overall means under the different support levels across all participants. If there were no or too few usable lifts from a subject under a specific condition (since the data has been omitted because of measurement artefacts), the data from this level of support were left out. However, the subject’s data under the remaining levels were used. The correlation coefficients *r*_*s*_ were classified as small (*r*_*s*_ = 0.10), medium (*r*_*s*_ = 0.30) or strong (*r*_*s*_ = 0.50) (Cohen, 1988). The significance level was set at *p* < 0.05. Since based on theoretical presumptions, a positive correlation between the level of support and the reduction of muscle activity was assumed, one-sided testing was performed.

To examine potential differences in the perceived rating of exertion for statistical significance as well, the RPE values achieved under the three main lifting conditions 1) without exoskeleton, 2) inactive exoskeleton (0% support level) and 3) active exoskeleton (100% support level) were compared in a similar way to the procedure regarding the muscle activity. However, since the RPE values were classified as ordinally scaled, a Friedman test was used. If a main effect could be observed, *post hoc* tests according to Dunn-Bonferroni were carried out afterwards to enable statements about the specific differences between the individual conditions. Again, the significance level was set at *p* < 0.05. All statistical analyses were carried out using the statistics software SPSS (Version 27.0, IBM^®^ SPSS^®^ Statistics).

## 3 Results

### 3.1 Impact of the lifting condition on MES activity

The data of all subjects (*n* = 14) could be considered for the investigation of the impact of the lifting condition on the MES activity as the corresponding intervals were free of visible artefacts. Data are presented as the mean (*M*) and standard deviation (SD) in % MVC.

**FIGURE 5:**
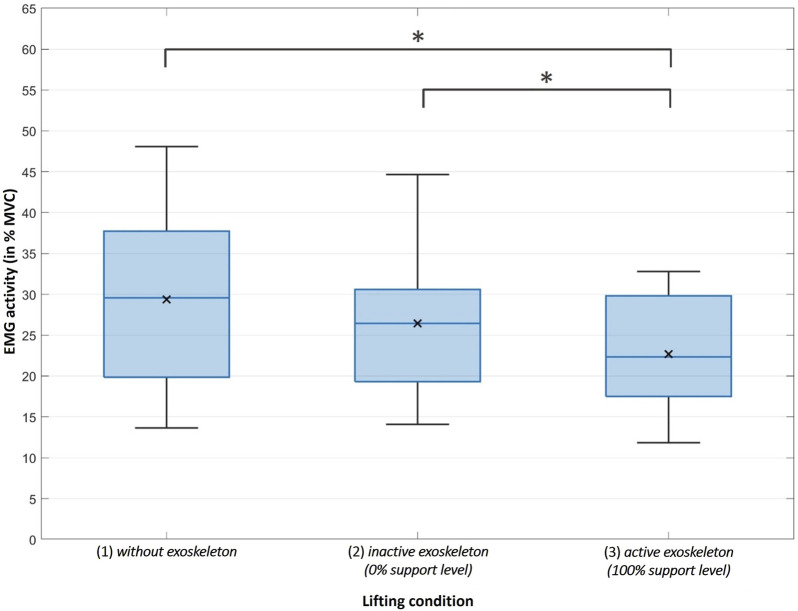
The coloured horizontal line within each boxplot represents the median RMS of the MES activity whereas the symbol x represents the mean RMS of the MES activity under the different main lifting conditions. The upper and lower borders of the boxplot represent the interquartile range and the whiskers extend to the largest or smallest RMS activity achieved (but reach maximum to 1.5 times the interquartile range). Extreme outliers (more than 1.5 times the interquartile range) would be shown separately (as a + sign) but did not occur in the three conditions compared. The horizontal bars with the symbol * thereby mark the conditions that differ significantly from each other (*p* < 0.05).

**FIGURE 6:**
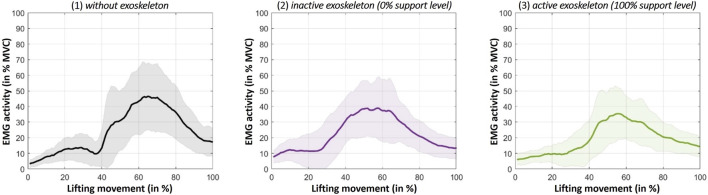
The solidly coloured curves represent the activity courses over the full lifting movement averaged over all subjects under the three compared main lifting conditions. The pale-coloured areas around the curves represent the standard deviation. The first increase in activity at the beginning of the lifting movement is due to the lowering movement of the upper body. The following larger increase is the phase of the actual lifting of the 15 kg box until the upright position is reached again (at 100%) and the lifting movement is completed.

The rmANOVA shows a significant influence of the lifting condition on MES activity (F_(2,26)_ = 18.431, *p* < 0.001, η_P_^2^ = 0.586). Bonferroni-corrected *post hoc* tests show significant differences specifically between the lifting conditions 1) without exoskeleton (*M* = 29.37, SD = 10.70) and 3) active exoskeleton (*M* = 22.70, SD = 7.59) (*p* < 0.001) as well as between 2) inactive exoskeleton and 3) active exoskeleton (*p* = 0.006) (Figure 5). This means that lifting with the active exoskeleton (100% support level) results in a significantly lower MES activity than lifting without exoskeleton or with the inactive exoskeleton (0% support level). The difference between lifting without exoskeleton and with the inactive exoskeleton (*M* = 26.45, SD = 8.85) does not turn out to be statistically significant (*p* = 0.056). The differences in muscle activity between the compared conditions mentioned before are also notable when looking at time-normalized courses of the EMG data (Figure 6).

### 3.2 Relation between the support level and MES activity

**FIGURE 7:**
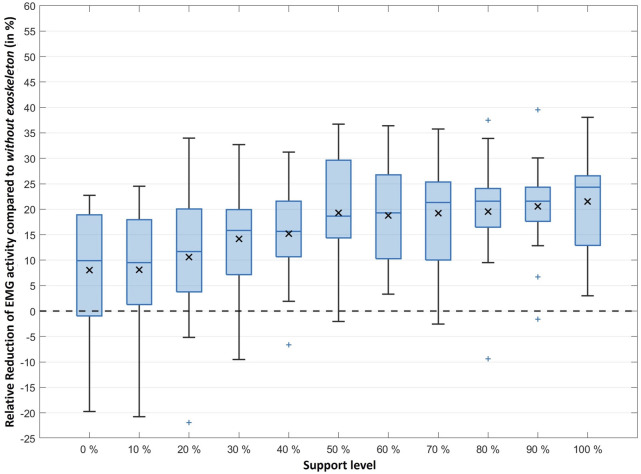
The coloured horizontal line within each boxplot represents the median reduction whereas the symbol x represents the mean reduction of the MES activity under the different support levels. The upper and lower borders of the boxplot represent the interquartile range and the whiskers extend to the largest or smallest reduction achieved (but reach maximum to 1.5 times the interquartile range). Extreme outliers (more than 1.5 times the interquartile range) are shown separately as a coloured + sign.

To examine the relationship between the exoskeleton’s support level and the MES activity, percentage differences relative to lifting without exoskeleton were calculated for each subject (for individual results see Supplementary Table S1). For this purpose, the non-normalized RMS values [mV] were used and compared, resulting in relative differences. On average, the use of the exoskeleton yielded a reduction of the MES activity by 8%–22% with increasing support levels compared to lifting without exoskeleton (Figure 7).

However, with low levels of support, a few individuals also experienced increased MES activity (negative percentage values, e.g., for subject 9 when using 0%–20% support) compared to lifting without exoskeleton. Subject 12 in particular is conspicuous, as most of its trials result in such negative percentage changes, but the activity data follow the general downward trend with increasing support levels.

To statistically investigate the relative reduction in MES activity with increasing support level, a Spearman correlation was performed for the levels of support and the mean percentage reductions obtained. For two subjects, no results were available for one of the support levels as the number of necessary repetitions was not reached due to measurement artefacts (missing values in Supplementary Table S1). Accordingly, only the data of 13 subjects were included for the affected levels (specifically 60% and 80% support level), whereas the data of all subjects could be included for all remaining levels. The Spearman correlation confirms the visual impression of a relationship between the support level and MES activity reduction (Figure 7) as a strong significant correlation can be seen regarding the mean values (*r*_*s*_ = 0.973, *p* < 0.001, *n* = 11, where *n* at this point represents the number of different support levels). In a second step, Spearman correlations were additionally carried out separately for the results of the individual subjects. A significant positive correlation was found for 11 of the 14 subjects (Table 1).

**TABLE 1:** Reduction in MES activity with increasing support levels examined for the individual subjects by Spearman correlations. At this point, *n* represents the number of different support levels for which values were available. A significant (*p* < 0.05) positive correlation was found for 11 of the 14 subjects and marked with the symbol *.

Subject	*r*_*s*_	*p*	*n*
1	0.309	*p* = 0.178	11
2	0.864	*p* < 0.001*	11
3	−0.018	*p* = 0.479	11
4	0.936	*p* < 0.001*	11
5	0.976	*p* < 0.001*	10
6	0.773	*p* = 0.003*	11
7	0.682	*p* = 0.010*	11
8	0.818	*p* = 0.001*	11
9	0.736	*p* = 0.005*	11
10	0.773	*p* = 0.003*	11
11	0.855	*p* < 0.001*	10
12	0.782	*p* = 0.002*	11
13	0.436	*p* = 0.090	11
14	0.982	*p* < 0.001*	11

### 3.3 Effect of the exoskeleton on the overall rating of perceived exertion (RPE)

**FIGURE 8:**
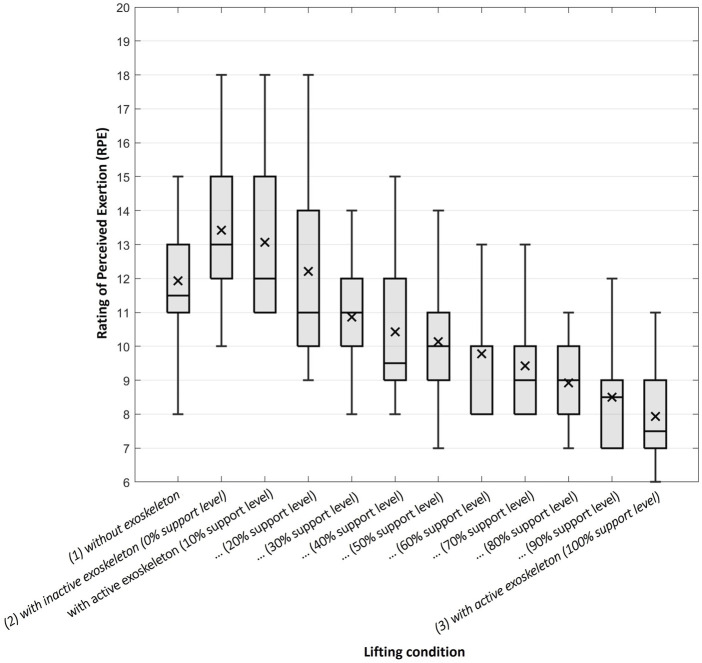
The horizontal line within each boxplot represents the median RPE whereas the symbol x represents the mean RPE under the several conditions. The upper and lower borders of the boxplot represent the interquartile range and the whiskers extend to the largest or smallest RPE stated (but reach maximum to 1.5 times the interquartile range). Extreme outliers (more than 1.5 times the interquartile range) would be shown separately (as a + sign) but did not occur regarding the RPE values.

A clear downtrend in perceived overall exertion with an increasing level of support can be seen in general (Figure 8). Comparing the results regarding the RPE under the three main lifting conditions using the Friedman test, a main effect can be found (Chi-Quadrat (2) = 21.28, *p* < 0.001, *n* = 14). The *post hoc* tests carried out afterwards show that when lifting with the 3) active exoskeleton (100% support level), the perceived load (*M* = 7.9, SD = 1.3) was significantly lower than when lifting 1) without exoskeleton (*M* = 11.9, SD = 1.9) (*z* = 1.214, *p*_*adjusted*_ = 0.004, effect size according to Cohen *r* = 0.324) or lifting with the 2) inactive exoskeleton (0% support level) (*M* = 13.4, SD = 2.5) (*z* = 1.571, *p*_*adjusted*_ < 0.001, effect size according to Cohen *r* = 0.42). Even though the general perceived rate of exertion turned out slightly higher when the subjects were lifting with the inactive exoskeleton compared to without exoskeleton, the corresponding difference is not statistically significant (*z* = −0.357, *p*_*adjusted*_ = 1).

## 4 Discussion

### 4.1 Classification of the results

Overall, the results of the current study (on average up to approx. 22% reduction in MES activity) lie within the range reported for the reduction in MES activity induced by the use of active exoskeletons (see review by Kermavnar et al. (2021)) and thus confirm their generally supportive effect.

As the present work is the first to solely focus on examining the impact of the Cray X exoskeleton (4th generation), we will compare the results in the following with findings of studies investigating rather similar types of exoskeletons. Furthermore, we focus on research using similar experimental setups and tasks to enable the comparison of corresponding results (Pesenti et al., 2021). Therefore, the most suitable studies seem to be those considering dynamic lifting movements too (instead of also commonly investigated static bending tasks) and which were conducted with an active back-supporting exoskeleton with a similar mode of action. These are, among others, studies including the so-called Robo-Mate exoskeleton, which provides assistive torque via electromotors at the user’s hip level too (Stadler et al., 2017; Huysamen et al., 2018; Toxiri et al., 2018; Lazzaroni et al., 2019). However, unlike the Cray X, it is at a prototype status and not commercially available. Huysamen et al. (2018) investigated the effect of the Robo-Mate exoskeleton on the peak activity of the MES and the perceived muscular effort while symmetric lifting and lowering weights from mid-shin height to waist height. No more detailed information on the lifting technique was given. They found a reduction in peak muscle activity of 12% MVC and 15% MVC when lifting 7.5 kg and 15 kg, respectively, compared to the lifting condition without exoskeleton. Since they looked at the peak values, higher results in % MVC were obtained. The mentioned reduction in % MVC corresponds to a relative reduction of activity compared to without exoskeleton by about 28%, thus showing similar effects to those found in our study. Regarding the perceived muscular effort, they report a corresponding reduction of approx. 10% (for 7.5 kg load) and 11% (for 15 kg load) for the trunk, using the Borg Category Ratio scale (CR-10). In contrast to the Cray X system, in this study, the Robo-Mate’s assistive strategy was solely based on the posture (torso inclination angle measured via an attached inertial measurement unit), which determined the assistive torque (Huysamen et al., 2018). A factor for the maximum torque could be selected within a certain range by the participants, but only before testing commenced. There are further studies involving the Robo-Mate which evaluate the impact of different control strategies. Next to the inclination-based control system, there are concepts including EMG-based and acceleration-based approaches (Toxiri et al., 2018; Koopman et al., 2019; Lazzaroni et al., 2019), or, like for an evolution of the Robo-Mate exoskeleton, the so-called XoTrunk, a constant torque strategy is used (specifically for carrying weights) (Poliero et al., 2020). An insight into the studies mentioned, regarding different control strategies of active exoskeletons and their effects on muscle activity when lifting or manually handling weight, can be found in the supplementary material (Supplementary Text S2).

Since there are no studies with an active exoskeleton where the effect of changing the level of support has been evaluated, no direct comparison of the current results with the state of research is possible. However, analogies can be found in a study conducted by Frost et al. (2009) with the so-called PLAD (Personal Lift Assistive Device), a passive exoskeleton. They used the exoskeleton under varying settings by manually adapting the stiffness of its elastic elements running parallel to the wearer’s spine and storing energy in form of deformation during the downwards phase of lifting and releasing it when going upwards, thereby changing the degree of support. Based on their results, an increase in the stiffness of the elastic elements is associated with a decrease in MES activity. This is generally consistent with the results of the present work, since in both cases, an increase in stiffness in the passive exoskeleton PLAD and an increase in support level in the active exoskeleton Cray X, a higher contribution to the extension moment can be assumed, thus reducing the needed muscular activation.

Furthermore, based on our results, we might assume that the observed reduction in MES activity may lead to a decrease in the MES force required for the lifting movement. We did not perform kinematic measurements of the lifting movement in this study. However, due to the same range of motion (defined by the given stoop technique) as well as a similar movement time (*p* = 0.162) of the lifting task with (*t* = 3.87 ± 0.63 s, 100% support level) and without exoskeleton (*t* = 4.00 ± 0.71 s), we can assume in a first approximation that the length change and the contraction velocity of the MES are similar under both conditions. If we continue to assume that the activation of the other muscles involved in the movement remains unchanged, one can estimate the MES force (*F = F*_*im*_ *· f*_*l*_ *· f*_*v*_ *· A*) using the product approach of classic Hill-type muscle models (Siebert and Rode, 2014), where *f*_*v*_ is the parameter for the force-velocity relation, *f*_*l*_ is the parameter for the force-length relation and *F*_*im*_ is the maximum isometric muscle force. If the parameters *F*_*im*_ *, f*_*l*_ and *f*_*v*_ remain unchanged, a reduction in muscle activation *A* leads to a reduction in muscle force. We are aware that this is just a first approximation and that further studies are necessary to measure the activation of all the muscles involved in the movement and analyse the kinematics of movement to, among others, make more precise statements about muscle force, e.g., using inverse dynamics in combination with multi-body models (Rupp et al., 2015). If the necessary muscle strength is reduced by providing a supportive torque when lifting, the mechanical load on the spine might be reduced (Näf et al., 2018; Kermavnar et al., 2021). Since cumulative mechanical loading of the lower back is associated with low back pain (Coenen et al., 2014), the exoskeleton could be useful in preventing MSD.

Lastly, next to a reduction in MES activity, we generally observed a decrease in the rate of perceived exertion with an increasing support level of the exoskeleton, resulting in a significantly lower RPE when lifting with the active exoskeleton (100% support level). However, a partial restriction of movement resulting from the tight-fitting straps and belts (to keep the spine in its natural shape), the general unfamiliar situation when wearing the exoskeleton as well as the additional dead weight might have influenced the RPE. This could explain the visually recognisable, though not statistically significant increase in RPE when lifting with the inactive exoskeleton (0% support level) compared to without exoskeleton (Figure 8). As the feeling of exertion can have multiple sources, the amount of MES activity does not solely determine the RPE. For example, Glinski et al. (2019) reported a significant reduction in activity without changes in RPE. However, when looking at the relation between RPE and the support levels across the whole range in our study, subjects assessed the exertion as almost linearly lower with increasing support levels, although they did not know under which level they were currently conducting the lifting movements. Concluding, the relief resulting from the exoskeleton’s support is clearly noticeable. This is also in agreement with an investigation by Schalk et al. (2022), examining the perception of exertion during the target-oriented use of multiple different exoskeletons. At this point, it might also be assumed that the muscular relief which was found for the MES in our study is present in other muscle groups too, explaining the perception is that distinct.

### 4.2 Limitations

Even though the MES is the most frequently investigated muscle when looking at the muscular effects of back-supporting exoskeletons (Kermavnar et al., 2021), the restriction to only this specific muscle is a limitation of our study. In future studies, other muscles (e.g., multifidus muscle, Wang et al. (2023)) should be taken into account to enable more general statements about the muscular effects of exoskeleton use. At this point, it should also be mentioned that despite the short familiarization period at the beginning of the study, the results only reflect the acute muscular effects of using a back-supporting exoskeleton in rather unexperienced users who had little to no expertise in using an exoskeleton. It might be assumed, for instance, that more experienced participants would have been more engaged with the exoskeleton and its supportive mode of action, which in turn may have manifested in even clearer results.

Apart from that, the perception of comfort or discomfort might have influenced the RPE results. Unfortunately, we did not record the parameter comfort. We only can state that none of the subjects actively reported any discomfort resulting from the exoskeleton’s assistance during the conduct of the study on their own. However, this does not necessarily mean that the level with the highest reduction in MES activity or perceived exertion is also the one they would actually prefer for real usage. At the workplace, users would probably select the level of support most suitable for the given conditions (e.g., weight to be lifted, body weight) based on a balance of comfort and reduction of exertion. Thus, subsequent investigations should vary the requirements (e.g., use different loads) and additionally examine how comfortable the assistance resulting from the different support levels is perceived by the subjects.

## 5 Conclusion

In this study, the effect of the commercially available active exoskeleton Cray X (4th generation) on the MES activity and RPE during lifting has been analysed. As many of the so far evaluated exoskeletons in literature are still at a prototype or early experimental stage, especially regarding active systems, and the exoskeleton market as a whole is still rather young, the results of our study could have an impact on the actual on-site use and acceptance of exoskeletons in commercial companies.

We found a significant reduction (approx. 22%) in MES activity when using the exoskeleton with maximum support level compared to lifting without exoskeleton. Regarding the RPE values, a significant reduction could be observed as well. MES activity and subjectively perceived exertion decreased, with an increase in the exoskeleton’s level of support. Thus, the exoskeleton seems to actively support the user noticeably when lifting heavy weights. However, no general recommendations can be made regarding the support level, even though the maximum level of support seems appropriate for the setup we have chosen, as the given conditions (e.g., load to be lifted, body weight, personal preferences) likely play a role here and therefore should always be taken into account. This once again highlights the significance of the control system of exoskeletons and how important the adjustability of the assistance is in order to facilitate versatile application.

## Data Availability

The raw data supporting the conclusion of this article will be made available by the authors, without undue reservation.

## Abbreviations

MSD, musculoskeletal disorders; MES, *M. erector spinae*/erector spinae muscle; EMG, electromyography; RPE, rating of perceived exertion; MVC, maximum voluntary contractions; RMS, root-mean-square; rmANOVA, one-way repeated measures analysis of variance; M, mean; SD, standard deviation.
