# Supplementary material for: Active exoskeleton reduces erector spinae muscle activity during lifting
Source: Front Bioeng Biotechnol. 2023 Apr 25;11:1143926. doi: 10.3389/fbioe.2023.1143926 (PMC10168292; doi:10.3389/fbioe.2023.1143926)
Supplement: Supplementary file 1 [file Table1.docx]

Supplementary Material

Active exoskeleton reduces erector spinae muscle activity during lifting

Tobias Walter^*^, Norman Stutzig, Tobias Siebert

*** Correspondence:** wtobias39@outlook.de

# Table S1

Relative reduction in the activity of the *M. erector spinae* compared to without exoskeleton (in %). The letter P stands for the term *subject*. Therefore, columns P1 to P14 represent the individual results of the fourteen subjects with regard to relative muscle activity reduction compared to lifting without exoskeleton. Positive values mean a reduction and negative values mean an increase in *M. erector spinae* activity. If a value is missing (-) this means that the minimum number of necessary repetitions was not reached due to measurement artefacts (two or more repetitions were affected by measurement artefacts and therefore excluded from further evaluation). Note that reduced EMG activity when using the inactive exoskeleton might be due to a form of passive support provided by the rigid structure of the exoskeleton and the elastic straps running across the gluteus to prevent slippage of the leg attachments.

|  | **Mean Reduction** | **8.05** | **8.11** | **10.59** | **14.20** | **15.19** | **19.31** | **18.77** | **19.24** | **19.56** | **20.54** | **21.51** |
| --- | --- | --- | --- | --- | --- | --- | --- | --- | --- | --- | --- | --- |
| **Relative Reduction of EMG activity compared to without exoskeleton per subject (in %)** | **P 14** | -2.40 | 4.87 | 10.07 | 17.19 | 10.85 | 19.24 | 19.74 | 24.01 | 25.71 | 24.15 | 26.59 |
|  | **P 13** | 18.33 | 24.53 | 21.78 | 19.90 | 21.61 | 22.18 | 25.17 | 25.33 | 21.76 | 21.33 | 25.96 |
|  | **P 12** | -19.72 | -20.72 | -21.88 | -9.53 | -6.66 | -2.01 | 5.29 | -2.53 | -9.41 | -1.62 | 3.00 |
|  | **P 11** | 20.18 | 13.04 | 20.04 | 19.68 | 29.49 | 29.61 | 30.76 | 35.77 | - | 30.06 | 33.60 |
|  | **P 10** | 5.78 | 2.19 | 5.55 | 12.18 | 1.92 | 10.15 | 14.37 | 6.91 | 17.98 | 17.61 | 17.69 |
|  | **P 9** | -3.36 | -5.68 | -0.31 | 10.11 | 10.69 | 15.82 | 9.84 | 6.39 | 17.72 | 12.86 | 12.93 |
|  | **P 8** | 14.27 | 14.87 | 13.32 | 15.66 | 16.18 | 21.84 | 14.84 | 21.17 | 19.76 | 24.34 | 24.58 |
|  | **P 7** | 1.23 | 1.29 | 3.75 | 2.50 | 10.64 | 7.98 | 10.39 | 16.28 | 12.68 | 6.72 | 9.21 |
|  | **P 6** | 2.44 | -5.48 | -5.16 | 0.80 | 9.76 | 14.79 | 3.32 | 10.00 | 9.54 | 18.89 | 11.70 |
|  | **P 5** | -0.93 | 5.98 | 9.57 | 7.14 | 15.36 | 14.36 | - | 21.48 | 21.99 | 27.61 | 33.18 |
|  | **P 4** | 14.06 | 17.97 | 15.41 | 16.00 | 20.77 | 18.11 | 19.26 | 22.85 | 21.61 | 23.57 | 25.43 |
|  | **P 3** | 22.75 | 22.65 | 24.49 | 30.25 | 15.90 | 30.54 | 28.47 | 30.29 | 33.89 | 21.82 | 15.18 |
|  | **P 2** | 21.17 | 20.39 | 33.95 | 32.68 | 31.23 | 36.75 | 36.41 | 33.40 | 37.50 | 39.51 | 38.08 |
|  | **P 1** | 18.90 | 17.65 | 17.70 | 24.21 | 24.88 | 30.98 | 26.17 | 18.05 | 23.56 | 20.78 | 24.08 |
|  |  | **0% support level** | **10% support level** | **20% support level** | **30% support level** | **40% support level** | **50% support level** | **60% support level** | **70% support level** | **80% support level** | **90% support level** | **100% support level** |
