# Supplementary material for: Active exoskeleton reduces erector spinae muscle activity during lifting
Source: Front Bioeng Biotechnol. 2023 Apr 25;11:1143926. doi: 10.3389/fbioe.2023.1143926 (PMC10168292; doi:10.3389/fbioe.2023.1143926)
Supplement: Supplementary file 2 [file DataSheet1.docx]

Supplementary Material

Active exoskeleton reduces erector spinae muscle activity during lifting

Tobias Walter^*^, Norman Stutzig, Tobias Siebert

*** Correspondence:** wtobias39@outlook.de

# Text S2

**Control strategies in active exoskeletons**

Toxiri et al. (2018) investigated the influence of different control strategies of the active exoskeleton Robo-Mate on the muscle activity of the MES when lifting. Besides the strategy based on trunk inclination (Huysamen et al., 2018), they implemented an EMG-based control strategy and a third strategy, a generalized combination of the first two ones. For the EMG-based strategy, the muscle activity of the forearm was measured by an EMG armband, based on the idea that the activity in the forearm muscles increases when grabbing and lifting a heavy object and then concomitantly a correspondingly higher level of support is required. As in the study by Huysamen et al. (2018), the subjects had to complete a lifting and lowering task with 7.5 kg as well as with 15 kg, using the different control strategies, without instructions on lifting technique or lifting speed. Toxiri et al. (2018) observed a reduction in the average activation profile as well as in the peak activation under all three strategies and for both loads. The peak activation was significantly reduced, ranging from 28% to 35%, compared to without exoskeleton. Shortly after, Koopman et al. (2019) published an article where additionally to the three control strategies different lifting techniques were used (free, squat and stoop lifting). They observed an average lumbar EMG reduction of approx. 13% MVC (averaged over all control strategies and lifting techniques) regarding back muscle peak activity when lifting a 15 kg heavy box. The observed reductions in muscle activity compared to lifting without exoskeleton were statistically significant, regardless of the control strategy or lifting technique used. Specifically, for the stoop technique (due to the similarity to our study), the peak activity was reduced from 68% MVC (without exoskeleton) to 57% MVC (exoskeleton with the inclination-based control strategy), 60% MVC (exoskeleton with EMG-based control strategy) and 58% MVC (exoskeleton with hybrid control strategy). Across the three different control strategies, this corresponds to a reduction of approx. 10% MVC, and hence, a relative reduction compared to stoop-lifting without exoskeleton of approx. 14%. In a recent study conducted by Poliero et al. (2020), using the so-called XoTrunk exoskeleton, which was derived from the Robo-Mate active trunk prototype (Di Natali et al., 2021; Toxiri et al., 2018), a constant torque strategy was implemented, in this case to support when carrying weights. Interestingly, the number of subjects who show a benefit from exoskeleton use increased as the carried weight increased. Even though their experimental results do not reveal a clear polarity of the data, there is a trend showing that MES activity is slightly reduced when carrying medium (8.2 kg) and higher weights (16.2 kg), whereas it seems that there is a slight increase in activity when carrying very low weight (1.2 kg). They assume that this might be due to adopted abdominal and back-extensor co-contraction, stiffening the upper body to counteract the constant extension torque of the exoskeleton and to regain stability when carrying very light weight. Since there are even further control strategies, e.g., based on the movement kinematics, as implemented by Lazzaroni et al. (2019) or in the case of the used exoskeleton Cray X and all strategies seem to have certain advantages and disadvantages, further research is needed here. At the same time, however, this shows how versatile active exoskeletons are, just by the possibility of controlling their actuators via the control system.

References

Di Natali, C., Chini, G., Toxiri, S., Monica, L., Anastasi, S., Draicchio, F., . . . Ortiz, J. (2021). Equivalent Weight: Connecting Exoskeleton Effectiveness with Ergonomic Risk during Manual Material Handling. *International Journal of Environmental Research and Public Health*, *18*(5). https://doi.org/10.3390/ijerph18052677

Huysamen, K., Looze, M. P. de, Bosch, T., Ortiz, J., Toxiri, S., & O'Sullivan, L. W. (2018). Assessment of an active industrial exoskeleton to aid dynamic lifting and lowering manual handling tasks. *Applied Ergonomics*, *68*, 125–131. https://doi.org/10.1016/j.apergo.2017.11.004

Koopman, A. S., Toxiri, S., Power, V., Kingma, I., van Dieën, J. H., Ortiz, J., & Looze, M. P. de (2019). The effect of control strategies for an active back-support exoskeleton on spine loading and kinematics during lifting. *Journal of Biomechanics*, *91*, 14–22. https://doi.org/10.1016/j.jbiomech.2019.04.044

Lazzaroni, M., Toxiri, S., Caldwell, D. G., Anastasi, S., Monica, L., Momi, E. de, & Ortiz, J. (2019). Acceleration-based Assistive Strategy to Control a Back-support Exoskeleton for Load Handling: Preliminary Evaluation. *IEEE … International Conference on Rehabilitation Robotics : [Proceedings]*, *2019*, 625–630. https://doi.org/10.1109/ICORR.2019.8779392

Poliero, T., Lazzaroni, M., Toxiri, S., Di Natali, C., Caldwell, D. G., & Ortiz, J. (2020). Applicability of an Active Back-Support Exoskeleton to Carrying Activities. *Frontiers in Robotics and AI*, *7*, 579963. https://doi.org/10.3389/frobt.2020.579963

Toxiri, S., Koopman, A. S., Lazzaroni, M., Ortiz, J., Power, V., Looze, M. P. de, . . . Caldwell, D. G. (2018). Rationale, Implementation and Evaluation of Assistive Strategies for an Active Back-Support Exoskeleton. *Frontiers in Robotics and AI*, *5*, 53. https://doi.org/10.3389/frobt.2018.00053
